# Recent advances in the management of dry age-related macular degeneration: A review

**DOI:** 10.12688/f1000research.10664.1

**Published:** 2017-03-09

**Authors:** Francesco Bandello, Riccardo Sacconi, Lea Querques, Eleonora Corbelli, Maria Vittoria Cicinelli, Giuseppe Querques

**Affiliations:** 1 Department of Ophthalmology, University Vita-Salute, IRCCS Ospedale San Raffaele, Milan, Italy; 2 Department of Ophthalmology, University of Verona, University hospital of Verona, Verona, Italy

**Keywords:** Age-related macular degeneration, Anti-inflammatory agents, Geographic atrophy, dry-AMD, Lipofuscin, Neuroprotective therapy, Nutritional Supplements, Stem cell-based therapy

## Abstract

Age-related macular degeneration (AMD), the most important cause of vision loss in elderly people, is a degenerative disorder of the central retina with a multifactorial etiopathology. AMD is classified in dry AMD (d-AMD) or neovascular AMD depending on the presence of choroidal neovascularization. Currently, no therapy is approved for geographic atrophy, the late form of d-AMD, because no treatment can restore the damage of retinal pigment epithelium (RPE) or photoreceptors. For this reason, all treatment approaches in d-AMD are only likely to prevent and slow down the progression of existing atrophy. This review focuses on the management of d-AMD and especially on current data about potential targets for therapies evaluated in clinical trials.

Numerous examinations are available in clinics to monitor morphological changes in the retina, RPE and choroid of d-AMD patients. Fundus autofluorescence and optical coherence tomography (OCT) are considered the most useful tools in the diagnosis and follow-up of d-AMD alterations, including the monitoring of atrophy area progression. Instead, OCT-angiography is a novel imaging tool that may add further information in patients affected by d-AMD.

Several pathways, including oxidative stress, deposits of lipofuscin, chronic inflammation and choroidal blood flow insufficiency, seem to play an important role in the pathogenesis of d-AMD and represent possible targets for new therapies. A great number of treatments for d-AMD are under investigation with promising results in preliminary studies. However, only few of these drugs will enter the market, offering a therapeutic chance to patients affected by the dry form of AMD and help them to preserve a good visual acuity. Further studies with a long-term follow-up would be important to test the real safety and efficacy of drugs under investigation.

## Introduction

Age-related macular degeneration (AMD) is the most important cause of vision loss in elderly people in developed countries ^1, 2^. Given that age is the primary risk factor for AMD, the prevalence and severity of this disease are likely to increase as human life expectancy increases ^3^. The exact pathophysiological mechanisms behind AMD remain to be determined, but certainly AMD is a multifactorial pathology, in which genetic and environmental risk factors play a crucial role ^4^. Early/intermediate stages of AMD, clinical conditions without overt functional loss, are characterized by deposition of drusen and/or retinal pigment epithelium (RPE) alterations in the macular area ^5^. In the late stages, the disease may progress to either geographic atrophy (GA) or neovascular AMD (n-AMD). The presence of choroidal neovascularization (CNV) is the hallmark of n-AMD that distinguishes this form from non-neovascular dry AMD (d-AMD). In the last years, the introduction in clinics of intravitreal injections of antivascular endothelial growth factor (anti-VEGF) drugs and the development of new therapies targeting vessel maturation and remodeling have revolutionized the natural history of the disease ^6– 8^. In contrast, no existing approved therapy for GA is available because no treatment is able to repair damaged RPE or photoreceptors. For this reason, all treatment approaches are only likely to slow down the progression of existing atrophy.

This review focuses on the management of d-AMD and especially on current data of studies and clinical trials about drugs that have already been evaluated or are under investigation in the management of dry AMD.

## Management of dry AMD: monitoring progression

The term “dry AMD” is commonly used to cover a range of fundus signs, including drusen and pigmentary changes to patchy areas of atrophy and GA ^9^. Reticular pseudodrusen represent an additional phenotype, associated with worse visual function from early stage, and an overall higher likelihood of progression to both forms of late AMD (n-AMD and GA) ^10– 12^. All these morphological findings of the retina, RPE and choroid are monitored by fundus photography, fundus autofluorescence (FAF), optical coherence tomography (OCT), infrared reflectance (IR) and optical coherence tomography angiography (OCT-A). Fundus photography has limited value in assessing and monitoring the progression of atrophic areas. FAF is currently considered as the gold standard in monitoring progression of atrophic areas; some authors suggest that FAF may also predict the rate of GA progression ^13, 14^. Structural OCT is currently extensively used in clinical practice as standard for d-AMD diagnosis and follow-up, as it allows a great visualization, measurement and monitoring of retinal layers, RPE, hyperreflective foci, GA areas and drusen ^15– 18^. OCT-A is a new noninvasive imaging tool able to characterize and quantify the vascular network in early, intermediate and advanced forms of d-AMD ^19– 21^. It has been demonstrated that in the early stages of the disease the choroidal layer shows dramatic alterations in its composition, with a predominance of stromal tissue on the vascular network ^19^. Although it is still an emerging technique, OCT-A is a promising imaging device that may add further information in patients affected by d-AMD and may provide support in relating structural and functional changes.

## Management of dry AMD: current therapeutic developments

Several pathways have been studied and related to the pathogenesis of d-AMD, including oxidative stress, deposits of lipofuscin, chronic inflammation (including complement activation), and choroidal blood flow insufficiency ^22^. A great number of treatments for d-AMD are under study. In this section, we analyze the main treatments under study by dividing therapeutic agents into six categories.

### Nutritional supplements

In recent years, there has been an enormous interest about nutrition and its relation to health. Many researchers demonstrated that food components are able to decrease the incidence of several diseases, including AMD. The AREDS study has shown that AREDS formula supplementation (a daily dose of 80 mg zinc oxide, 2 mg cupric oxide, 15 mg β-carotene, 500 mg vitamin C and 400 IU vitamin E) was effective in certain categories of patients affected by d-AMD, significantly reducing the risk of AMD progression ^23^. In particular, these results were achieved in patients with high-risk, using the late stage disease as the primary endpoint. Whether AREDS formula supplementation also has a beneficial effect in patients affected by the earliest stages of the disease is unknown ^23^. Also in GA patients these results were not confirmed; partially, this was due to the relative small sample size of GA patients included in the study ^23^. However, patients affected by a specific form of GA, involving the central area of the retina, were found to benefit from AREDS supplementation formula because they showed lower rates of progression to n-AMD, similarly to patients affected by a moderate stage of d-AMD ^24^. Since, β-carotene increases the incidence of lung cancer in smokers ^25^, a following study (AREDS2) evaluated the effect of β-carotene elimination from the original AREDS formula supplementation ^26^. AREDS2 demonstrated that β-carotene elimination or lower-dose zinc did not influence the progression to late AMD ^26^. However, a great incidence of lung cancer was recorded in patients treated with AREDS formula supplementation compared with patients treated with AREDS2 formula supplementation, mostly in former smokers ^26^.

A new active area of research is the association between the vitamin supplements and genetic profile: the interest was based on evidence that showed that genetic risk profile of a patient may influence the benefit of vitamin supplements ^27^. The results on this specific area will be available in the next years. However, there is general agreement that the AREDS and AREDS2 supplementation have a healthy effect mainly for their antioxidative action, and that they will play a great role in the treatment of d-AMD patients for a long period of time.

### Anti-inflammatory therapy

Chronic inflammation is thought to be crucial for AMD pathogenesis ^3, 28^. Currently, corticosteroids are being investigated for their antiangiogenic and antiinflammatory effect. Iluvien (Alimera Sciences, Alpharetta, GA, USA) is a sustained-release formulation of fluocinolone acetonide, just approved for the treatment of diabetic macular edema (DME), which could slow the progression of GA. A total of 40 patients affected bilaterally by GA were recruited in a phase II study (NCT00695318) ^29^. The study was completed, but the results are not yet available.

The histopathological identification of different complement complexes in patients with GA and the presence of variations in genes encoding complement proteins clarify several treatment strategies to study the contribution of systemic complement in the pathogenesis of the disease ^30, 31^. Although different complement inhibitors are being studied to treat GA, none has been approved yet or has proven to be effective.

POT-4 (Potentia Pharmaceuticals, Louisville, KY, USA; ALcon, Hünenberg, Switzerland) is a C3 inhibitor administered by intravitreal injection, with 6 months duration of action. A phase I clinical trial (NCT00473928) ^32^ was completed without safety concerns and a phase II clinical trial would be required to prove the safety and efficacy of this drug in d-AMD.

ARC1905 (Zimura; Ophthotech Corp., Princeton, NJ, USA) is an anti-C5 aptamer targeting C5, which has completed a phase I trial (NCT00950638) ^33^. Plans for initiating a phase II/III trial of ARC1905 are reported to be under way ( http://www.ophthotech.com/product-candidates/arc1905/).

Another drug targeting C5 is Eculizumab (Soliris; Alexon Pharmaceuticals, Cheshire, CT, USA). The COMPLETE study showed no reduction of GA progression by Eculizumab, but the low luminance deficit at baseline was significantly correlated with the progression of GA over 6 months ^34, 35^.

Lampalizumab (FCFD4514S; Genentech/Roche, San Francisco, CA, USA) is a humanized monoclonal antibody targeting complement factor D in the alternative complement pathway. The Lampalizumab phase II clinical trial (NCT02288559) ^36^ was the first study to demonstrate a positive effect in slowing growth of GA through complement inhibition. Two on-going phase III trials, Chroma (NCT02247479) ^37^ and Spectri (NCT02247531) ^38^ are under way to investigate the safety and efficacy of 10 mg Lampalizumab injections every 4 or 6 weeks vs sham injections.

Sirolimus (Rapamycin; MacuSight/Santen, Union City, CA, USA) is a macrolide immunosuppressive agent with antiinflammatory, antiangiogenic and antifibrotic activity. It was generally well tolerated, but no evidence of efficacy has been shown ^39^.

Glatiramer acetate (Copaxone; Reva Pharmaceuticals, Kfar-Saba, Israel) has been studied for its immunomodulatory effect altering T-cell differentiation in GA treatment. A phase I study (NCT00541333) ^40^ has demonstrated reduction of the drusen area in patients with drusen after weekly subcutaneous Glatiramer injections over 12 weeks. A phase II/III study (NCT00466076) is underway ^41^.

In addition, amyloid-beta may play a role in AMD progression. RN6G (Pfizer, New York, NY, USA) and GSK933776 (GlaxoSmithKline, Brentford, UK), humanized monoclonal antibodies targeting amyloid-beta have been studied in phase II clinical trials (NCT01577381 and NCT01342926, respectively) ^42, 43^, but results are not available.

### Neuroprotective therapy

Another interesting area under development is neuroprotection. There are two drugs under investigation: ciliary neurotrophic factor-501 (CNFT) and Brimonidine.

CNFT, a member of the IL-6 cytokine family, has been shown to protect photoreceptors in animal models ^44^. Neurotech Pharmaceuticals (Cumberland, RI, USA) developed a well-tolerated intraocular encapsulated cell technology (ECT), which when combined with CNTF in a sustained-release platform (NT-501), releases the substance for more than one year ^45^. A randomized, double-masked, phase II trial (NCT00447954) ^46^ studied the 2-year results of the NT-501 implant in GA patients with promising results. In total, 51 patients were randomized and treated by high-dose or low-dose NT-501 implants or sham treatment. Zhang *et al*. ^47^ showed a dose-dependent stabilization of visual acuity, evaluated as a loss of <15 letters on the ETDRS chart, in high-dose patients (96.3%) versus low-dose (83.3%) and sham treatment (75%) at 12-month evaluation. The stabilization of visual acuity was related with a retinal thickness increase at structural OCT.

Brimonidine, a member of α-2 agonists, which is frequently used in glaucoma patients, has also demonstrated a neuroprotective effect on retinal cells in animal models ^48, 49^. A multicenter, phase II, double-masked, randomized study (NCT00658619) ^50^ evaluated the efficacy and the safety of Brimonidine administered by an intravitreal biodegradable implant (Allergan, Irvine, CA, USA). The study evaluated the changes of GA area and BCVA in 119 patients with bilateral GA, randomly divided and treated by 200 or 400 μg of Brimonidine or a sham therapy every 3 months through month 21. The results did not show reliable data and for this reason a second multicenter trial (NCT02087085) ^51^ is currently ongoing. The primary outcome measure of this trial is the GA area change from baseline to 24-month evaluation in the 311 study eyes treated by 400 µg Brimonidine implant or sham treatment. The estimated study completion date is March 2019.

### Lipofuscin and visual cycle inhibitors

The rationale for using visual cycle inhibitors in the treatment of GA is the documented phototoxic and proinfiamamtory effect of lipofuscin accumulated at the sites of RPE atrophy in patients with GA ^52^.

Fenretinide (Sirion Therapeutics, Tampa, FL, USA) is a synthetic retinoid that competitively prevents the uptake of retinol by the RPE with downregulation of visual cycle. A phase II clinical trial (NCT00429936) ^53^ demonstrated that 100 mg and 300 mg daily Fenretinide did not reduce the growth rate of GA, but patients seemed to tolerate it well.

Emixustat (ACU-4489; Acucela, Seattle, WA, USA) is a non-retinoid visual cycle modulator of the isomerase (RPE65) preventing conversion of all-trans-retinol to 11-cis-retinalin in the RPE, with minor accumulation of lipofuscin. A phase IIa trial (NCT01002950) ^54^ showed a biological effect in GA eyes. Phase II/III study (NCT01802866) ^55^ was completed, but results are not available.

### Choroidal blood flow restoration agents

The choroid is diminished in thickness in older age patients and for this reason a new target therapy in d-AMD could lead to the restoration of a higher choroidal blood flow ^56^. Choroidal circulation plays an important role in providing nutrients and removing wastes from the RPE and retina layers ^57^. Many vasodilators are currently under investigation in clinical trials with the rationale that the use of these drugs may increase the blood flow of the choroid, and thus may delay the progression of d-AMD.

A phase 3, multicenter, controlled, randomized study (NCT00619229) ^58^ proved that Alprostadil (UCB Pharma, Berkshire, UK) was superior to placebo treatment in patients affected by d-AMD ^59^. Patients treated with Alprostadil showed a best-corrected visual acuity superior of 0.94 lines compared with patients treated by placebo after 3 months, increasing to 1.51 lines at 6-month follow-up. However, further trials are strongly recommended to evaluate the long-term effects and safety of Alprostadil, and to understand the role that this drug could play in d-AMD therapy.

A small pilot trial (NCT01922128) ^60^ studied a new vasodilator called MC-1101, and demonstrated not only a safe and well tolerated profile for topical administration, but also a choroidal blood flow increase. MC-1101 has also shown an anti-inflammatory and an antioxidant profile. The safety and efficacy of MC-1101 will be evaluated by a randomized phase II/III trial (NCT02127463) ^61^ that is currently ongoing and includes 60 patients affected by mild to moderate d-AMD.

Moxaverine, a nonselective phosphodiesterase inhibitor, showed contradictory results in different studies: Schmidl *et al*. ^62^ reported that oral administration of Moxaverine is not effective in increasing choroidal blood flow, while Resh *et al*. ^63^ and Pemp *et al*. ^64^ demonstrated that intravenous administrated Moxaverine increases choroidal blood flow compared with placebo. These different results may be due to the different mode of administration, but further studies are necessary to investigate the clinically efficacy of Moxaverine in patients affected by d-AMD.

Sildenafil (Viagra; Pfizer Inc, New York, NY, USA) is a known vasodilator, but its role in the treatment of d-AMD is not clear. Metelitsina *et al*. ^65^ reported that this drug was not effective in improving the choroidal blood flow of the fovea in patients affected by AMD.

### Stem cell-based therapy

Stem cell therapy represents a promising new approach for AMD. Evidence suggests that RPE and photoreceptors are primarily affected in GA, their transplantation seems to be an interesting therapeutic option ^66^. Thus, human pluripotent stem cells, embryonic (hESC) or induced (iPSC) are currently being investigated in clinical trials for AMD ^67– 69^.

However, stem-cell-based therapy carries a long-term and multi-disciplinary approach. Therefore, the pros and cons of therapy must be analyzed in order to fully develop these new attractive approaches.

## Conclusions

Geographic atrophy, the late form of d-AMD, is a progressive disease and no treatment is approved at the moment. Nevertheless, there are currently many trials underway with the aim of finding an effective drug in preventing the enlargement of the atrophy and to avoid d-AMD patients to progress to a more devastating form of the disease, and to maintain a good visual function. Probably, many drugs will prove ineffective for AMD and only a few will be available in clinical practice.

In this review, we focused on current data about potential targets for therapies that seem to play a crucial role in the progress of d-AMD, but the pathogenesis of the disease remains unclear. Future studies should focus on understanding all mechanisms connected to d-AMD and develop other approaches in the therapy of this disease.

Some of the drugs here described have been shown to be potentially effective in preliminary studies, and these are probably the ones that have more chance to be really effective in d-AMD patients and that will enter the market earlier. Nevertheless, first it will be important to prove the efficacy and the safety of the drugs currently investigated with long-term follow-up. Only after that we will have therapeutics to offer to our patients to help them to maintain their visual acuity.
