# Supplementary material for: Recent advances in the management of dry age-related macular degeneration: A review
Source: F1000Res. 2017 Mar 9;6:245. [Version 1] doi: 10.12688/f1000research.10664.1 (PMC5428517; doi:10.12688/f1000research.10664.1)
Supplement: Peer review history

# Peer review: Referee response for version 1

**DOI:** 10.5256/f1000research.11493.r22527

**Published:** 2017-05-09

**Authors:** Kamron N. Khan

**Affiliations:** 1 Section of Ophthalmology and Neuroscience, Leeds Institute of Biomedical and Clinical Sciences (LIBACS), University of Leeds, Leeds, UK; 2 NIHR Moorfields Biomedical Research Centre, London, UK; 3 Leeds Teaching Hospitals NHS Trust, Leeds, UK

Thank you for asking me to review this work. This is a clearly set out and easy to read manuscript, covering all major avenues of current research in non-neovascular AMD. I only have a couple of minor comments.

1. Perhaps the title could be altered to better reflect the manuscript content: “Therapeutic strategies under current investigation for dry age-related macular degeneration”?
2. I think the manuscript could be improved by expanding the section on “stem cells”.
3. A couple more trials may be worthy of mention (a) Oracea Phase 2/3 trial and (b) Drusen clearance with laser - Laser Intervention in Early Age-Related Macular Degeneration Study (LEAD).

I have read this submission. I believe that I have an appropriate level of expertise to confirm that it is of an acceptable scientific standard.

# Peer review: Referee response for version 1

**DOI:** 10.5256/f1000research.11493.r20863

**Published:** 2017-03-22

**Authors:** Igor Kozak

**Affiliations:** 1 Moorfield Eye Hospital Centre, Abu Dhabi, United Arab Emirates

The authors present a concise and yet comprehensive review on current therapies and ongoing clinical trials for non-exudative (dry) age-related macular degeneration. The authors list and comment on current trials registered at government website. As such, this represents a useful update for ophthalmologists in this area. Few minor comments for the authors:

 1.       I would suggest to expand the second paragraph dealing with associations between vitamin supplements and genetic profile under Nutritional Supplements section. While some studies suggest genotypic influence on clinical response to vitamin supplementation, there are opposing studies as well ^1^. Nice recent review by Rowan & Taylor (2016) ^2^ is also worth mentioning.

 2.       Apart from therapies as part of registered clinical trials there are few investigation running independently such as testing oral trimetazine (anti-ischemic agent with cytoprotective effects) by Institut de Recherches Internationales Servier; neuroprotective agent tandospirone by Alcon Research; oral crocetin; oral curcumin; or intravitreal LFG316 by Novartis (NCT01527500) and oral doxycycline (Oracea)(NCT01782989).

 3.       For section on stem cell-based therapy I would update literature on recent (even though not fully convincing) study by Schwartz SD *et al* (2016) ^3^ or the same author in Lancet 2015.

I have read this submission. I believe that I have an appropriate level of expertise to confirm that it is of an acceptable scientific standard.

## References

[1] ChewEYKleinMLClemonsTEAgrónERatnapriyaREdwardsAOFritscheLGSwaroopAAbecasisGRAge-Related Eye Disease Study Research Group: No clinically significant association between CFH and ARMS2 genotypes and response to nutritional supplements: AREDS report number 38. *Ophthalmology*.2014;121(11) : 10.1016/j.ophtha.2014.05.008 2173-80 10.1016/j.ophtha.2014.05.008 24974817PMC4253656

[2] RowanSTaylorA: Gene-Diet Interactions in Age-Related Macular Degeneration. *Adv Exp Med Biol*.2016;854: 10.1007/978-3-319-17121-0_14 95-101 10.1007/978-3-319-17121-0_14 26427399

[3] SchwartzSDTanGHosseiniHNagielA: Subretinal Transplantation of Embryonic Stem Cell-Derived Retinal Pigment Epithelium for the Treatment of Macular Degeneration: An Assessment at 4 Years. *Invest Ophthalmol Vis Sci*.2016;57(5) : 10.1167/iovs.15-18681 ORSFc1-9 10.1167/iovs.15-18681 27116660
